# Relation between risk of dysphagia and oral intake level in cancer patients

**DOI:** 10.1590/2317-1782/e20240100en

**Published:** 2025-02-10

**Authors:** Alexia Diovana Fernandes da Rocha, Monalise Costa Batista Berbert, Vera Beatris Martins

**Affiliations:** 1 Programa de Pós-graduação em Residência Multiprofissional Integrada em Saúde, Onco-hematologia, Universidade Federal de Ciências da Saúde de Porto Alegre – UFCSPA - Porto Alegre (RS), Brasil.; 2 Departamento de Fonoaudiologia, Universidade Federal de Ciências da Saúde de Porto Alegre – UFCSPA - Porto Alegre (RS), Brasil.; 3 Serviço de Fonoaudiologia, Santa Casa de Porto Alegre – SCPA - Porto Alegre (RS), Brasil.

**Keywords:** Speech, Language and Hearing Sciences, Dysphagia, Deglutition Disorders, Neoplasms, Medical Oncology

## Abstract

**Objective:**

To relate the self-perceived risk of dysphagia with the level of oral intake in hospitalized oncology patients.

**Methods:**

This cross-sectional study had a convenience sample of adults and older adults diagnosed with cancer and hospitalized in an oncology hospital in southern Brazil. Data on sex, age, length of hospitalization, comorbidities, oncological diagnosis, treatment, and feeding route were obtained from the participants' medical records. The level of oral intake was classified using the Functional Oral Intake Scale (FOIS), and the risk of dysphagia was identified using the Eating Assessment Tool (EAT-10). The relationship between these variables was analyzed using Spearman's correlation coefficient.

**Results:**

The study included 60 participants – 42 with solid tumors and 18 with hematological tumors; 35 females (58.3%) and 25 males(41.7%), with a mean age of 58.5 ± 13.1 years. Of these, 56 exclusively used the oral route for feeding (93.3%), and 18 were at risk of dysphagia (30%). Older patients were at higher risk for dysphagia than adults (p-value = 0.020). EAT-10 scores (median = 0; IQR = 0–4) were significantly inversely correlated (RHO = -0.463; p-value = 0.000) with FOIS classifications (N: level 2 = 2; level 3 = 2; level 4 = 2; level 5 = 12; level 7 = 42).

**Conclusion:**

The study found that lower EAT-10 scores corresponded to higher FOIS levels. In other words, the lower the risk of dysphagia, the lower the susceptibility to using alternative feeding routes.

## INTRODUCTION

Cancer is a complex disease considered the greatest public health problem worldwide^(1)^. Population aging, along with behavioral, environmental, and structural changes, are associated with the increasing incidence and mortality of cancer globally^(1,2)^. In Brazil, the estimated number of new cancer cases for the 2023–2025 triennium is 704,000, with non-melanoma skin cancer predicted to be the most common, followed by breast, prostate, colon and rectum, lung, and stomach cancer^(1)^.

Both the disease and its treatment can lead to significant and even debilitating complications in the person's life^(2)^. Oncology patients commonly experience dysphagia, associated with various factors, including the effects of the tumor itself, as well as surgical resection, chemotherapy, and radiotherapy^(3)^. Although this symptom is more frequent in head and neck cancers, it can also occur in other types of tumors^(3,4)^.

Dysphagia indicates an alteration in the process of moving solids or liquids from the oral cavity to the stomach^(3)^. The persistence of this difficulty can lead to dehydration, malnutrition, aspiration pneumonia, and even death. Its impacts negatively affect the social, psychological, and economic aspects of the patient’s life, as well as their quality of life^(5)^.

Early detection of dysphagia is crucial to ensure safe oral intake and adequate nutrition, reducing the risk of laryngotracheal aspiration and improving the patient’s overall clinical condition^(6,7)^. Dysphagia screening tools are quick to administer, non-invasive, may be self-assessed, and aim to identify this difficulty early, facilitating referral for swallowing evaluation by a speech-language-hearing pathologist^(7)^.

The use of dysphagia screening tools has previously been described in patients diagnosed with neoplasms, particularly in cases of head and neck tumors^(5,6,8-11)^. Considering that oncology patients may be at risk for dysphagia, it is important to relate their perceptions of swallowing difficulties to clinical speech-language-hearing assessment results, aiming to achieve safe feeding and good adherence to treatment. Hence, this study aimed to relate the self-perceived risk of dysphagia to the level of oral intake in hospitalized oncology patients.

## METHOD

This cross-sectional, quantitative study with a convenience sample was conducted at a reference oncology hospital in southern Brazil. The research was approved by the institution's Research Ethics Committee under evaluation report no. 6.035.026. All participants signed an informed consent form.

The sample consisted of individuals aged 18 years or older with an oncological diagnosis, hospitalized in the inpatient wards designated for the Brazilian Unified Health System (SUS) at a philanthropic hospital between May and June 2023. The sample size was estimated based on the number of beds in the inpatient units. Exclusion criteria were exclusive use of an alternative feeding route, poor general clinical condition preventing participation in the study, and absence of data in the electronic medical record.

Data on sex, age, length of hospitalization, comorbidities, oncological diagnosis, treatment, and feeding route were obtained from the participants' electronic medical records. The protocol for collecting this information was specifically developed for the study. The same speech-language-hearing pathologist conducted the entire assessment and data collection process.

The level of oral intake was classified using the Functional Oral Intake Scale (FOIS) after evaluating swallowing with different food consistencies. It classifies the person’s amount of oral intake (food and liquids) into seven specific levels^(12)^ – levels 1 to 3 indicate degrees of feeding that depend on an alternative feeding route (with or without oral intake), while levels 4 to 7 indicate degrees of feeding exclusively by oral route, with or without modifications, adaptations, and compensatory maneuvers in feeding^(8,12)^.

The risk of dysphagia was identified using the Eating Assessment Tool (EAT-10), a self-assessment instrument based on the person's perception of their swallowing^(13,14)^, with 10 items related to functioning, emotional impact, and physical symptoms caused by swallowing problems^(14)^. Each item is rated on a scale from 0 to 4, according to the difficulties experienced, where 0 indicates no perceived problem and 4 indicates a severe perceived problem^(5)^. A score ≥ 3 points indicates that the patient is at risk for dysphagia and should be referred for swallowing evaluation by a speech-language-hearing pathologist^(5,13)^.

The statistical analysis was performed using the Statistical Package for the Social Sciences (IBM SPSS Statistics for Windows, Version 25.0. Armonk, NY: IBM Corp.), with a 5% significance level (p ≤ 0.05). Qualitative variables were presented through absolute and relative frequencies. Quantitative variables were presented through mean and standard deviation, and median and interquartile range when asymmetrical. Normality was verified using the Shapiro-Wilk test. The Mann-Whitney, Kruskal-Wallis, and Spearman's correlation coefficient tests analyzed EAT-10 and FOIS scores. The chi-square and Fisher’s exact tests were used for analyses involving dysphagia risk and level of oral intake.

## RESULTS

The study included 60 hospitalized oncology patients – 35 females (58.3%) and 25 males (41.7%), with a mean age of 58.5 ± 13.1 years. The data regarding the participants’ clinical and demographic characteristics are presented in Table 1.

**Table 1:** Clinical and demographic characteristics of the sample

Variables	Distribution	
	N	%
**Age group**		
Adults (18-59 years)	27	45
Older adults (≥ 60 years)	33	55
**Location of tumors**		
Lymphatic and/or blood system	17	28.3
Digestive system	16	26.7
Reproductive system	10	16.7
Endocrine system	1	1.7
Head and neck	4	6.7
Bones	3	5
Skin	3	5
Breast	3	5
Lungs	3	5
**Treatment modality**		
Surg.	16	26.7
RT	1	1.7
CTX	13	21.7
BMT	1	1.7
CTX + Surg.	9	15
CTX + RT	3	5
RT + Surg.	3	5
Surg. + BMT	1	1.7
CTX + BMT	1	1.7
CTX + RT + Surg.	4	6.7
None	8	13.3
**Breathing**		
Room air	55	91.7
Room air with metal tracheostomy	2	3.3
Room air with O_2_ supply	3	5
**Walking**		
Yes	49	81.7
No	11	9.3
**Feeding support**		
Yes	3	5
No	57	95

**Caption:** Surg. = surgery; CTX = Chemotherapy; RT = Radiotherapy; BMT = bone marrow transplantation

Forty-two patients were diagnosed with solid tumors (71.7%) and 18 with hematological tumors (28.3%). Regarding the neoplasm treatment modalities, 31 patients received one treatment modality (51.7%), 17 received two (28.3%), four received three (6.7%), and eight did not undergo any treatment (13.3%).

The median length of hospitalization was 7.5 days (IQR = 3.2 - 15; minimum = 2 days, maximum = 83 days). Twenty-seven patients did not report comorbidities associated with neoplasia (45%), 25 had at least one comorbidity (41.7%), and eight had two or more comorbidities (13.4%).

Regarding feeding, 56 patients exclusively used the oral route (93.3%), and four used the oral route partially, complemented by an alternative feeding route (6.6%). The main aspect to be analyzed regarding dentition was the presence or absence of dental elements in the mastication process. Thus, the sample was divided into the presence of dental elements (natural teeth and/or dental prosthesis) and edentulism (complete absence of dental elements). Table 2 describes the data on the speech-language-hearing clinical findings.

**Table 2:** Speech-language-hearing clinical findings

Variables	Distribution	
	N	%
**Speech-language-hearing complaints**		
Voice	7	11.7
Swallowing	7	11.7
Voice + Swallowing	2	3.3
Voice + Hearing	1	1.7
None	43	71.7
**Dentition**		
With dental elements	58	96.7
Edentulism	2	3.3
**Oral hygiene**		
Adequate	52	86.7
Inadequate	8	13.3
**Oral diet (consistency)**		
Normal or soft	42	70
Pureed	12	20
Liquidized	4	6.7
Postoperative or totally liquid	2	3.3
**Speech-language-hearing procedure**		
Therapy	19	31.7
Discharge	41	68.3

Regarding the risk of dysphagia, the median EAT-10 score was 0 points (IQR = 0 - 4; minimum = 0, maximum = 25). In the analysis of the oral intake level using the FOIS (median = 7; IQR = 5 - 7), 93.3% of the sample did not use an alternative feeding route, and 70% had an unrestricted oral route (level 7). In this study, no participants were classified in FOIS level 1 (nothing by mouth) or level 6 (total oral diet with multiple consistencies without special preparation, but with specific food limitations). The data on the risk for dysphagia and the level of oral intake are presented in Table 3.

**Table 3:** Risk for dysphagia (EAT-10) and oral intake level (FOIS)

Variables	Distribution	
	N	%
**EAT-10 Classification**		
Presence of risk for dysphagia (≥ 3 points)	18	30
Absence of risk for dysphagia (≥ 3 points)	42	70
**FOIS Classification**		
Level 2	2	3.3
Level 3	2	3.3
Level 4	2	3.3
Level 5	12	20
Level 7	42	70

Caption: Level 2 = Tube dependent with minimal attempts of food or liquid; Level 3 = Tube dependent with consistent oral intake of food or liquid; Level 4 = Total oral diet of a single consistency; Level 5 = Total oral diet with multiple consistencies, but requiring special preparation or compensations; Level 7 = Total oral diet with no restrictions

**Figure 1:**
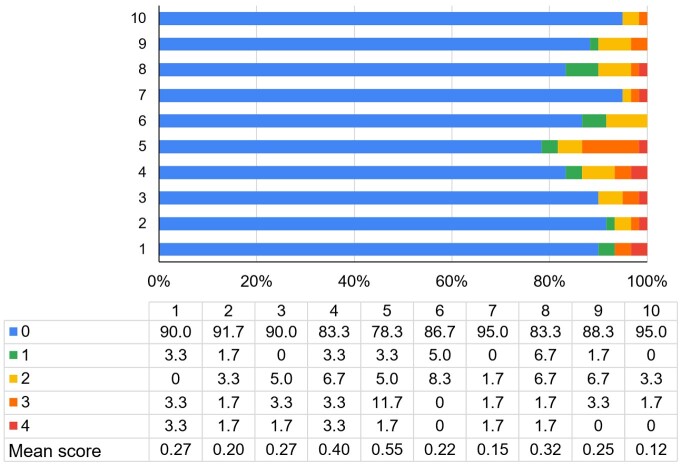
Mean and percentage scores in EAT-10 items

Item 5 of the EAT-10, which describes the need for extra effort to swallow medication, presented the highest mean score compared to the other items (0.55 points), with 21.7% of participants reporting some degree of difficulty (1 to 4 points). Among the patients at risk for dysphagia, 55.6% reported some degree of difficulty swallowing medication. Figure 1 provides a detailed breakdown of the EAT-10 item scores.

Caption: EAT-10 items: 1 - My swallowing problem has caused me to lose weight; 2 - My swallowing problem interferes with my ability to go out for meals; 3 - Swallowing liquids takes extra effort; 4 - Swallowing solids takes extra effort; 5 - Swallowing pills takes extra effort; 6 - Swallowing is painful; 7 - The pleasure of eating is affected by my swallowing; 8 - When I swallow food sticks in my throat; 9 - I cough when I eat; 10 - Swallowing is stressful

This study found a significant inverse correlation between the EAT-10 score and the FOIS classification, using Spearman's correlation coefficient (RHO = -0.463; p-value = 0.000). This finding means that the higher the EAT-10 score, the lower the level on the FOIS scale.

The risk of dysphagia was present in 33.3% of patients with solid tumors and 22.2% of patients with hematological tumors. No significant association was found between tumor type and location regarding the risk of dysphagia (p-value = 0.389; p-value = 0.316) and the use of an alternative feeding route (p-value = 1.000; p-value = 0.790), respectively. Similarly, the treatment modality was not related to the risk of dysphagia (p-value = 0.719) or the use of an alternative feeding route (p-value = 0.612).

The older patient group scored higher on the EAT-10 (p-value = 0.025) and were at a higher risk for dysphagia (p-value = 0.020) than the adult group – 77.8% of patients at risk for dysphagia were older adults. No relationship was found between age groups and the use of an alternative feeding route (p-value = 0.620).

## DISCUSSION

This study showed that the self-perceived risk of dysphagia in oncology patients aligns with the classification of oral intake level (use or not of an alternative feeding route) through clinical swallowing assessment. Dysphagia screening tools based on the patient's self-perception are essential for detecting early and evaluating how patients perceive the impact of swallowing difficulties on their lives, promoting better adherence to therapy and more effective outcomes, reducing complications, and improving quality of life^(6-8)^.

Currently, most studies in the literature investigating dysphagia in oncology patients focus on head and neck neoplasms^(5,6,8-11)^. Swallowing changes are frequent in these types of tumors due to their location, surgical sequelae, and radiotherapy and chemotherapy side effects^(15)^. This study has a unique sample, as it included patients with different types of tumors other than head and neck cancer, since dysphagia also affects these individuals, regardless of the location^(2,3)^.

Older patients (≥ 60 years) and females were more prevalent, which corroborates a study with oncology patients that observed a higher prevalence of females (51%) and a mean age of 59 years^(16)^. There may be diverging demographic characteristics compared to studies with head and neck cancer patients, as this type of tumor is more prevalent in older individuals (≥ 65 years) and males^(8-10)^.

Solid tumors were more prevalent than hematological tumors in the sample, corroborating the incidence data in the literature^(1)^. Regarding tumor location, those in the lymphatic and blood systems, as well as in the digestive system, were more frequent. Although these tumors do not always directly affect the structures responsible for swallowing, their treatment can cause symptoms that trigger dysphagia, such as mucositis, xerostomia, and odynophagia^(17,18)^. In esophageal tumors, in addition to the previously described manifestations, obstruction is a common complication, which can prevent the passage of food bolus to the stomach, causing chest pain and lower dysphagia^(19)^.

The most prevalent treatment modality in this sample was surgery, which can influence the development of swallowing problems. However, few participants underwent procedures in the upper respiratory tract and/or upper digestive tract. Chemotherapy was the second most frequent treatment modality, with mucositis commonly occurring in patients due to toxicity, which can hinder the process of oral feeding^(17,18)^. Head and neck radiation can affect swallowing muscles and cause sequelae that affect the biomechanics of swallowing^(15)^ – although few patients in this sample underwent radiotherapy. The characteristics regarding tumor location and treatment may explain the lack of relationship between oncology treatment type, dysphagia risk, and oral intake level in this study.

Enteral or parenteral nutritional support is common in oncology patients due to the risk of malnutrition caused by the disease's prognosis or its treatment’s side effects^(20,21)^. Orofacial complications, such as tooth loss and inadequate oral hygiene, are also frequent in this population, especially in patients with head and neck tumors, clinically unstable patients, or those in the terminal stage^(22,23)^. The findings of this study indicate that the low frequency of alternative feeding route use, the presence of dental elements, and adequate oral hygiene may be explained by clinical stability and the few patients with head and neck neoplasms.

This study showed that 30% of participants were at risk for dysphagia, corroborating research in which 54.4% of patients with different types of tumors reported some form of dysphagia-related symptom, with it being more common in head and neck tumors and less prevalent in breast cancer^(16)^. Another study with solid tumors outside the head and neck region and upper digestive tract identified dysphagia risk using the EAT-10 in 19% of the sample^(24)^. On the other hand, the risk for dysphagia assessed with the EAT-10 increases significantly in the population with head and neck tumors, with a prevalence of this risk ranging from 54.9% to 72.2% of patients^(17,25)^. Research on dysphagia in patients with tumors outside the head and neck region is still scarce in the literature, although they also suffer from swallowing issues^(2,3,16,24)^, thus supporting the population studied here.

Most patients in this sample were exclusively fed orally (FOIS 4-7) and without dietary restrictions (FOIS 7), which corroborates a study with the same population, showing that 96.1% were exclusively fed orally, of whom 56.7% did not need to make dietary adaptations or restrictions^(26)^. The prevalence of exclusive oral intake without restrictions is lower in patients with head and neck tumors than the general oncology population, as described in a study that observed that 59% of these patients were exclusively fed orally (FOIS ≥ 4)^(8)^. Another study with this same population identified that only 16.7% were on an unrestricted oral diet (FOIS 7), while 77.8% were on a full oral diet with adaptations, compensations, and/or restrictions (FOIS 5 or 6)^(25)^. The study patients’ oral intake condition can be justified by the sample being representative of the general oncology population.

The inverse correlation between the EAT-10 score and the FOIS classification in this study means that the lower the self-perceived risk of dysphagia, the better the oral intake level (without using an alternative feeding route or restrictions/adaptations in the oral diet). Therefore, it is believed that speech-language-hearing assessment should consider the patient's self-perception of eating.

The Spearman coefficient also found an inverse correlation between the level of oral intake (FOIS) and the self-perception of dysphagia severity (EAT-10) in patients with head and neck cancer^(8,9,11,25)^. As for patients with tumors outside the head and neck and upper digestive tract region, it was found that 21% had a risk for dysphagia (EAT-10 ≥ 3 points or complaints of difficulty swallowing and/or chewing) and had dysphagia confirmed through clinical swallowing evaluation using different criteria, one of them being FOIS < 7^(24)^. No studies were found in the literature that related the EAT-10 and FOIS findings in patients with different types of neoplasms, as in this study.

The item in the EAT-10 where patients scored the highest degree of difficulty was related to making extra effort to swallow medications (Figure 1), with a higher prevalence in those at risk for dysphagia. This finding corroborates a study that applied the EAT-10 to non-oncological individuals without complaints of dysphagia, in which 17% reported some degree of difficulty swallowing medications, whereas 72% of participants at risk for dysphagia reported this difficulty^(27)^. Other studies with healthy individuals also found similar results, with prevalence ranging from 10.4% to 32%^(28,29)^. This data is important because individuals with difficulty swallowing medications may discontinue their use or resort to inappropriate techniques to manage these difficulties, which can affect the medication’s effectiveness^(28,30)^.

It is understood that the presence of organic diseases such as stroke, neurodegenerative diseases, and neoplasms can further increase the prevalence of dysphagia among older people^(30,31)^. In this study, the risk for dysphagia was more prevalent in older adults, corroborating a study with hospitalized patients, which observed an association between older age and higher risk for dysphagia (EAT-10) – 61% of patients classified as at risk for dysphagia were 60 years or older^(32)^. Also, dysphagia in patients with laryngeal and hypopharyngeal cancer was more frequent in those aged 65 years or older^(33)^.

This study included individuals with different types of neoplasms, not only head and neck tumors, and had good representation regarding sex, age, and age groups (adults and older adults). As a limitation of the study, it used a representative sample of a patient profile from a reference center for oncological treatment, with its size estimated by the number of available beds. Additionally, the decision was made not to perform objective swallowing assessments due to the characteristics of the service's routine. Future studies should involve other populations, larger sample sizes, and complementary swallowing assessments with objective swallowing tests.

## CONCLUSION

This study demonstrated that the lower the EAT-10 score, the higher the FOIS level in hospitalized oncological patients. In other words, the lower the risk for dysphagia, the lower the likelihood of needing an alternative feeding route.

This inverse correlation between the risk of dysphagia and the level of oral intake suggests that the better the patient's self-perception of swallowing, the better the oral feeding condition. This finding highlights the importance of using self-assessment tools for swallowing in the general cancer population, as these screening instruments enable the quick identification of individuals who need specialized swallowing evaluation, promoting early diagnosis and intervention.
