# Supplementary material for: Relation between risk of dysphagia and oral intake level in cancer patients
Source: Codas. 2025 Feb 10;37(2):e20240100. doi: 10.1590/2317-1782/e20240100en (PMC11813178; doi:10.1590/2317-1782/e20240100en)
Supplement: Translation

# Translation: Relação entre risco de disfagia e nível de ingestão oral em pacientes oncológicos

**DOI:** 10.1590/2317-1782/e20240100pt

**Authors:** Alexia Diovana Fernandes da Rocha, Monalise Costa Batista Berbert, Vera Beatris Martins

**Affiliations:** 1 Programa de Pós-graduação em Residência Multiprofissional Integrada em Saúde, Onco-hematologia, Universidade Federal de Ciências da Saúde de Porto Alegre – UFCSPA - Porto Alegre (RS), Brasil.; 2 Departamento de Fonoaudiologia, Universidade Federal de Ciências da Saúde de Porto Alegre – UFCSPA - Porto Alegre (RS), Brasil.; 3 Serviço de Fonoaudiologia, Santa Casa de Porto Alegre – SCPA - Porto Alegre (RS), Brasil.

**Keywords:** Fonoaudiologia, Disfagia, Transtornos de Deglutição, Neoplasias, Oncologia

## Abstract

**Objetivo:**

Relacionar o risco de disfagia autopercebido com o nível de ingestão oral em pacientes oncológicos hospitalizados.

**Método:**

Estudo transversal, com amostra por conveniência composta por indivíduos adultos e idosos, com diagnóstico de câncer e internados em um hospital oncológico do sul do Brasil. Os dados sobre sexo, idade, tempo de internação, comorbidades, diagnóstico oncológico, tratamento e via de alimentação, foram obtidos dos prontuários dos participantes. O nível de ingestão oral foi classificado pela Functional Oral Intake Scale (FOIS), o risco de disfagia foi identificado pelo Eating Assessment Tool (EAT-10) e a relação entre eles foi feita por meio do coeficiente de correlação de Spearman.

**Resultados:**

Foram incluídos 60 participantes (42 tumores sólidos e 18 tumores hematológicos), sendo 35 do sexo feminino (58,3%) e 25 do sexo masculino (41,7%), com média de idade de 58,5 ± 13,1 anos. Destes, 56 se alimentavam por via oral exclusiva (93,3%) e 18 apresentavam risco de disfagia (30%). Os idosos apresentaram maior risco para disfagia em comparação aos adultos (p-valor = 0,020). A relação entre a pontuação do EAT-10 (mediana 0; IQR = 0 - 4) e a classificação da FOIS (N: nível 2 = 2; nível 3 = 2; nível 4 = 2; nível 5 = 12; nível 7 = 42) demonstrou correlação inversa significativa (RHO = -0,463; p-valor = 0,000).

**Conclusão:**

Observou-se que quanto menor a pontuação no EAT-10, maior o nível da FOIS. Ou seja, quanto menor o risco para disfagia, menor a suscetibilidade ao uso de via alternativa de alimentação.

## INTRODUÇÃO

O câncer é uma doença complexa e é considerada o maior problema de saúde pública no mundo^(1)^. O envelhecimento populacional e as mudanças comportamentais, ambientais e estruturais estão relacionados com o aumento na incidência e na mortalidade por câncer no cenário mundial^(1,2)^. Para o Brasil, a estimativa para o triênio de 2023-2025 aponta que ocorrerão 704 mil casos novos de câncer no país, sendo o câncer de pele não melanoma estimado como o mais incidente, seguido pelos cânceres de mama, próstata, cólon e reto, pulmão e estômago^(1)^.

Tanto a doença quanto o seu tratamento podem causar complicações significativas e até mesmo debilitantes para a vida do indivíduo^(2)^. Os pacientes oncológicos comumente apresentam disfagia, que está associada a diversos fatores, incluindo os efeitos do próprio tumor, bem como da ressecção cirúrgica, da quimioterapia e da radioterapia^(3)^. Apesar deste sintoma ser mais frequente em neoplasias de cabeça e pescoço, ele pode ocorrer em outros tipos de tumores^(3,4)^.

A disfagia indica a presença de alteração no processo de passagem de sólidos ou de líquidos da cavidade oral até o estômago^(3)^. A persistência desta dificuldade pode resultar em desidratação, desnutrição, pneumonia aspirativa e até morte. Os seus impactos afetam negativamente os aspectos sociais, psicológicos e econômicos da vida do paciente, bem como a sua qualidade de vida^(5)^.

A detecção precoce da disfagia é importante para garantir a segurança da ingestão via oral e a nutrição adequada, reduzindo o risco de aspiração laringotraqueal e melhorando a condição clínica geral do paciente^(6,7)^. Os instrumentos de rastreio para disfagia são de rápida aplicação, não invasivos, podem ser auto avaliativos e têm como objetivo a identificação precoce desta dificuldade, propiciando o encaminhamento para avaliação da deglutição com fonoaudiólogo^(7)^.

O uso de instrumentos de rastreio para disfagia já foi descrito previamente em pacientes diagnosticados com neoplasias, principalmente nos casos de tumores de cabeça e pescoço^(5,6,8-11)^. Considerando que pacientes oncológicos podem apresentar risco para disfagia, é importante relacionar suas percepções sobre as dificuldades na deglutição com os resultados da avaliação clínica fonoaudiológica, visando alcançar uma alimentação segura e uma boa adesão ao tratamento. Neste contexto, o presente estudo tem como objetivo relacionar o risco de disfagia autopercebido com o nível de ingestão oral em pacientes oncológicos hospitalizados.

## MÉTODO

Trata-se de um estudo transversal, quantitativo, com amostragem por conveniência, desenvolvido em um hospital oncológico de referência da região sul do Brasil. A pesquisa foi aprovada pelo Comitê de Ética em Pesquisa da instituição, sob o parecer nº 6.035.026. Todos os participantes assinaram o Termo de Consentimento Livre e Esclarecido (TCLE).

A amostra foi composta por indivíduos maiores de 18 anos, com diagnóstico oncológico e que estavam internados nas enfermarias destinadas ao Sistema Único de Saúde (SUS) de um hospital filantrópico, no período entre maio e junho de 2023. O tamanho amostral foi estimado pelo número de leitos nas unidades de internação. Foram considerados critérios de exclusão: via alternativa de alimentação exclusiva, más condições do estado clínico geral que impedissem de responder à pesquisa e ausência de dados em prontuário eletrônico.

Os dados relacionados ao sexo, idade, tempo de internação, comorbidades, diagnóstico oncológico, tratamento e via de alimentação, foram obtidos dos prontuários eletrônicos dos participantes. O protocolo para coleta destas informações foi elaborado exclusivamente para o estudo. Todo o processo de avaliação e coleta de dados foi conduzido pela mesma fonoaudióloga.

O nível de ingestão oral foi classificado pela *Functional Oral Intake Scale* (FOIS) após avaliação da deglutição com diferentes consistências alimentares. A FOIS é uma escala que classifica em níveis específicos a quantidade de ingestão por via oral de um indivíduo (alimentos e líquidos)^(12)^. A escala consiste em sete níveis: os níveis de 1 a 3 indicam os graus de alimentação que dependem da via alternativa de alimentação (com ou sem via oral) e os níveis de 4 a 7 apontam os graus de alimentação exclusivamente por via oral, com ou sem modificações, adaptações e manobras compensatórias na alimentação^(8,12)^.

O risco para disfagia foi identificado por meio do *Eating Assessment Tool* (EAT-10), que é um instrumento de autoavaliação a partir da percepção do indivíduo sobre sua deglutição^(13,14)^.O instrumento consiste em 10 itens sobre funcionalidade, impacto emocional e sintomas físicos causados por problemas na deglutição^(14)^. Cada item deve ser respondido numa escala de 0 a 4, de acordo com dificuldades vivenciadas, em que zero indica que não há problema autopercebido e 4 indica um grande problema autopercebido^(5)^. Uma pontuação ≥ 3 pontos indica que o paciente apresenta risco para disfagia e deve ser encaminhado para avaliação da deglutição com fonoaudiólogo^(5,13)^.

A análise estatística foi realizada no software Statistical Package for Social Sciences (IBM SPSS Statistics for Windows, Version 25.0. Armonk, NY: IBM Corp.), com valor de significância de 5% (p ≤ 0.05). As variáveis qualitativas foram apresentadas por meio de frequência absoluta e relativa. As variáveis quantitativas foram apresentadas por meio de média e desvio-padrão, e de mediana e intervalo interquartil, quando assimétricas. A normalidade foi verificada pelo teste Shapiro-Wilk. Foram aplicados os testes Mann-Whitney, Kruskal-Wallis e coeficiente de correlação de Spearman para as análises com os escores EAT-10 e FOIS. Os testes Qui-Quadrado e Exato de Fisher foram aplicados para as análises com risco para disfagia e com nível de ingestão oral.

## RESULTADOS

O estudo foi composto por 60 pacientes oncológicos hospitalizados, sendo 35 do sexo feminino (58,3%) e 25 do sexo masculino (41,7%), com média de idade de 58,5 ± 13,1 anos. Os dados referentes às características clínicas e demográficas dos participantes estão apresentados na Tabela 1.

**Tabela 1:** Características clínicas e demográficas da amostra

Variáveis	Distribuição	
	N	%
**Grupo etário**		
Adulto (18-59 anos)	27	45
Idoso (≥ 60 anos)	33	55
**Localização dos tumores**		
Sistema linfático e/ou sanguíneo	17	28,3
Sistema digestivo	16	26,7
Sistema reprodutivo	10	16,7
Sistema endócrino	1	1,7
Cabeça e pescoço	4	6,7
Ossos	3	5
Pele	3	5
Mama	3	5
Pulmões	3	5
**Modalidade de tratamento**		
CX	16	26,7
RT	1	1,7
QT	13	21,7
TMO	1	1,7
QT + CX	9	15
QT + RT	3	5
RT + CX	3	5
CX + TMO	1	1,7
QT + TMO	1	1,7
QT + RT + CX	4	6,7
Nenhuma	8	13,3
**Respiração**		
Ar ambiente	55	91,7
Ar ambiente com TQT metálica	2	3,3
Ar ambiente com suporte O2	3	5
**Deambulação**		
Sim	49	81,7
Não	11	9,3
**Auxílio na alimentação**		
Sim	3	5
Não	57	95

Legenda: CX = Cirurgia; QT = Quimioterapia; RT = Radioterapia; TMO = Transplante de medula óssea; TQT = Traqueostomia

Quarenta e dois pacientes foram diagnosticados com tumores do tipo sólido (71,7%) e 18 com tumores do tipo hematológico (28,3%). Em relação à modalidade de tratamento para as neoplasias, 31 pacientes receberam uma modalidade de tratamento (51,7%), 17 receberam duas (28,3%), 4 receberam três (6,7%) e 8 não realizaram tratamento (13,3%).

A mediana em relação à internação hospitalar foi de 7,5 dias (IQR = 3,2 - 15; mínimo = 2 dias, máximo = 83 dias). Vinte e sete pacientes não referiram comorbidades associadas a neoplasia (45%), 25 possuíam pelo menos uma (41,7%) e 8 possuíam duas ou mais (13,4%).

Em relação à alimentação, 56 pacientes se alimentavam por via oral exclusiva (93,3%) e 4 por via oral parcial complementada pela via alternativa de alimentação (6,6%). O principal aspecto a ser analisado a respeito da dentição, foi a presença ou não de elementos dentários no processo de mastigação, sendo assim, a amostra foi dividida em: elementos dentários presentes (dentes próprios e/ou prótese dentária) e edentulismo (ausência total de elementos dentários). A Tabela 2 descreve os dados sobre os achados clínicos fonoaudiológicos.

**Tabela 2:** Achados clínicos fonoaudiológicos

Variáveis	Distribuição	
	N	%
**Queixas fonoaudiológicas**		
Voz	7	11,7
Deglutição	7	11,7
Voz + Deglutição	2	3,3
Voz + Audição	1	1,7
Nenhuma	43	71,7
**Dentição**		
Elementos dentários presentes	58	96,7
Edentulismo	2	3,3
**Higiene oral**		
Adequada	52	86,7
Inadequada	8	13,3
**Dieta VO (consistência)**		
Normal ou branda	42	70
Pastosa	12	20
Pastosa liquidificada	4	6,7
Líquida completa ou pós-operatória	2	3,3
**Conduta fonoaudiológica**		
Terapia	19	31,7
Alta	41	68,3

**Legenda:** VO = Via oral

Quanto ao risco para disfagia, a mediana de pontuação do EAT-10 foi de 0 pontos (IQR = 0 - 4; mínimo = 0, máximo = 25). Na análise do nível de ingestão oral pela FOIS (mediana = 7; IQR = 5 - 7), 93,3% da amostra não utilizava via alternativa de alimentação, sendo que 70% apresentava via oral sem restrições (nível 7). Neste estudo, não tivemos participantes classificados no nível 1 (nada por via oral) e nem no nível 6 (via oral total com múltiplas consistências, sem necessidade de preparo especial ou compensações, porém com restrições alimentares) da FOIS. Os dados sobre o risco para disfagia e o nível de ingestão oral estão dispostos na Tabela 3.

**Tabela 3:** Risco para disfagia (EAT-10) e nível de ingestão oral (FOIS)

Variáveis	Distribuição	
	N	%
**Classificação no EAT-10**		
Presença de risco para disfagia (≥ 3 pontos)	18	30
Ausência de risco para disfagia (< 3 pontos)	42	70
**Classificação na FOIS**		
Nível 2	2	3,3
Nível 3	2	3,3
Nível 4	2	3,3
Nível 5	12	20
Nível 7	42	70

**Legenda:** Nível 2 = dependente de via alternativa e mínima via oral de algum alimento ou líquido; Nível 3 = dependente de via alternativa com consistente via oral de alimento ou líquido; Nível 4 = via oral total de uma única consistência; Nível 5 = via oral total com múltiplas consistências, porém com necessidade de preparo especial ou compensações; Nível 7 = via oral sem restrições

O item 5 do EAT-10, que descreve a situação referente à necessidade de fazer força para engolir remédios, apresentou a maior média de pontos em comparação aos demais itens (0,55 pontos), sendo que 21,7% dos participantes pontuaram algum grau de dificuldade (1 a 4 pontos). Dos pacientes com risco para disfagia, 55,6% referiram algum grau de dificuldade para engolir remédios. A Figura 1 apresenta detalhadamente a relação de pontos dos itens do EAT-10.

**Figura 1:** Pontuação dos itens do EAT-10 por média e por porcentagem

**Legenda:** Itens do EAT-10: 1 - Meu problema para engolir me faz perder peso; 2 - Meu problema para engolir não me deixa comer fora de casa; 3 - Preciso fazer força para beber líquidos; 4 - Preciso fazer força para engolir comida (sólidos); 5 - Preciso fazer força para engolir remédios; 6 - Dói para engolir; 7 - Meu problema para engolir me tira o prazer de comer; 8 - Fico com comida presa/entalada na garganta; 9 - Eu tusso quando como; 10 - Engolir me deixa estressado

No presente estudo, observou-se correlação inversa significativa entre a pontuação do EAT-10 e a classificação da FOIS, por meio do coeficiente de correlação de Spearman (RHO = -0,463; p-valor = 0,000). Este achado significa que quanto maior a pontuação no EAT-10, menor o nível na escala FOIS.

O risco para disfagia estava presente em 33,3% dos pacientes com tumores do tipo sólido e em 22,2% dos pacientes com tumores do tipo hematológico. Não houve associação significativa entre o tipo e a localização do tumor, tanto em relação ao risco para disfagia (p-valor = 0,389; p-valor = 0,316), quanto ao uso de via alternativa de alimentação (p-valor = 1,000; p-valor = 0,790), respectivamente. Da mesma forma, a modalidade de tratamento não apresentou relação com o risco para disfagia (p-valor = 0,719) e o uso de via alternativa de alimentação (p-valor = 0,612).

O grupo de pacientes idosos obteve uma pontuação mais alta no EAT-10 (p-valor = 0,025) e demonstrou um risco maior para disfagia (p-valor = 0,020) em comparação ao grupo de adultos, sendo que 77,8% dos pacientes com risco para disfagia eram idosos. Não houve relação entre os grupos etários e o uso de via alternativa de alimentação (p-valor = 0,620).

## DISCUSSÃO

O presente estudo mostrou que o risco de disfagia autopercebido em pacientes oncológicos concorda com a classificação do nível de ingestão oral (uso ou não de via alternativa de alimentação) por meio da avaliação clínica da deglutição. Os instrumentos de triagem para disfagia a partir da opinião do próprio paciente são essenciais para detecção precoce e para avaliação de como os pacientes percebem o impacto das dificuldades na deglutição em sua vida, promovendo melhor adesão à terapia e resultados mais efetivos, bem como redução de complicações e melhora da qualidade de vida^(6-8)^.

Atualmente, a maioria dos estudos presentes na literatura que investigam a disfagia em pacientes oncológicos são voltados às neoplasias de cabeça e pescoço^(5,6,8-11)^. As alterações na deglutição são frequentes nestes tipos de tumores por conta da localização, bem como das sequelas cirúrgicas e dos efeitos colaterais da radioterapia e da quimioterapia^(15)^. Este estudo apresenta um diferencial em relação à amostra, pois foram incluídos pacientes com diferentes tipos de tumores, não só em região de cabeça e pescoço, visto que a disfagia também acomete estes indivíduos, independentemente da localização^(2,3)^.

Os pacientes idosos (≥ 60 anos) e do sexo feminino foram mais prevalentes, corroborando com um estudo com pacientes oncológicos, que observou maior prevalência de pessoas do sexo feminino (51%) e uma média de idade de 59 anos^(16)^. Possíveis discordâncias nas características demográficas em relação aos estudos com pacientes com câncer de cabeça e pescoço podem ocorrer, pois este tipo de tumor é mais prevalente em indivíduos idosos (≥ 65 anos) e do gênero masculino ^(8-10)^.

Os tumores sólidos foram mais prevalentes na amostra quando comparados aos tumores hematológicos, corroborando os dados de incidência presentes na literatura^(1)^. Em relação a localização do tumor, foram mais frequentes aqueles no sistema linfático e sanguíneo, bem como no sistema digestivo. Apesar destes tumores nem sempre afetarem diretamente as estruturas responsáveis pela deglutição, o tratamento para a doença pode causar sintomas que promovem o surgimento da disfagia, como mucosite, xerostomia e odinofagia**^(^** ^17,18)^. Em tumores no esôfago, além das manifestações já descritas, a obstrução é uma complicação comum, podendo impedir a passagem do bolo alimentar para o estômago, ocasionando dor torácica e disfagia baixa^(19)^.

A modalidade de tratamento mais prevalente nesta amostra foi a cirurgia, que pode influenciar no desenvolvimento de problemas na deglutição, porém poucos participantes realizaram o procedimento no trato respiratório superior e/ou no trato digestivo alto. A quimioterapia foi a segunda modalidade de tratamento mais frequente, sendo comum a ocorrência de mucosite nos pacientes por conta da toxicidade, o que pode dificultar o processo de alimentação por via oral**^(^** ^17,18)^. A radiação em região de cabeça e pescoço pode causar mudanças na musculatura envolvida na deglutição, além de sequelas que afetam a biomecânica da deglutição^(15)^, porém nesta amostra poucos pacientes realizaram tratamento radioterápico. As características referentes à localização do tumor e ao tratamento, podem explicar a ausência de relação entre tipo de tratamento oncológico, risco de disfagia e nível de ingestão oral neste estudo.

O suporte nutricional enteral ou parenteral é comum em pacientes oncológicos, por conta do risco de desnutrição causado pelo próprio prognóstico da doença ou pelos efeitos colaterais do seu tratamento^(20,21)^. Nesta população também são frequentes complicações orofaciais, como perdas dentárias e higiene oral inadequada, principalmente em pacientes com tumores de cabeça e pescoço, clinicamente instáveis ou em fase de terminalidade^(22,23)^. Diante dos achados deste estudo, acredita-se que a baixa frequência de uso de via alternativa de alimentação, bem como a presença de elementos dentários e a higiene oral adequada, podem ser justificados pela estabilidade clínica e pelo baixo número de pacientes com neoplasias de cabeça e pescoço.

O presente estudo demonstrou que 30% dos participantes apresentaram risco para disfagia, corroborando com um estudo em que 54,4% dos pacientes com diferentes tipos de tumor relataram algum tipo de sintoma relacionado à disfagia, sendo mais comum em tumores de cabeça e pescoço e menos prevalentes em câncer de mama^(16)^. Outro estudo com tumores sólidos fora da região de cabeça e pescoço e trato digestivo alto, identificou a presença de risco para disfagia por meio do EAT-10 em 19% da amostra^(24)^. Por outro lado, o risco para disfagia avaliado com o EAT-10, aumenta consideravelmente na população com tumores de cabeça e pescoço, sendo possível identificar uma prevalência deste risco em 54,9% a 72,2% dos pacientes^(17,25)^. A investigação da disfagia em pacientes com neoplasias fora da região de cabeça e pescoço ainda é escassa na literatura, apesar destes indivíduos também sofrerem com problemas na deglutição^(2,3,16,24)^, respaldando, assim, a população aqui estudada.

A maioria dos pacientes nesta amostra alimentava-se por via oral exclusiva (FOIS 4-7) e sem restrições na dieta (FOIS 7), corroborando com um estudo com a mesma população, que demonstrou que 96,1% estavam se alimentando por via oral exclusiva e, destes, 56,7% não precisavam realizar adaptações ou restrições na dieta^(26)^. Em pacientes com tumores de cabeça e pescoço, observa-se menor prevalência de via oral exclusiva sem restrições quando comparado a população oncológica em geral, como descrito em um estudo que observou que 59% destes pacientes alimentavam-se exclusivamente por via oral (FOIS ≥ 4)^(8)^. Outro estudo com esta mesma população, identificou que somente 16,7% estavam com dieta via oral sem restrições (FOIS 7), enquanto 77,8% estavam com via oral total com adaptações, compensações e/ou restrições (FOIS 5 ou 6)^(25)^. A condição da ingestão oral dos pacientes deste estudo pode ser justificada pela amostra ser representativa da população oncológica.

Neste estudo, a correlação inversa entre a pontuação do EAT-10 e a classificação na FOIS significa que quanto menor o risco de disfagia autopercebido, melhor o nível de ingestão oral (sem uso de via alternativa de alimentação ou restrições/adaptações na dieta oral). Por isso, acredita-se que a autopercepção do paciente em relação a sua alimentação deve ser levada em consideração no processo avaliativo fonoaudiológico.

Em pacientes com câncer de cabeça e pescoço também foi observada correlação inversa entre o nível de ingestão oral (FOIS) e a autopercepção da severidade da disfagia (EAT-10), por meio do coeficiente de Spearman^(8,9,11,25)^. Em pacientes com tumor fora da região de cabeça e pescoço e trato digestivo alto, observou-se que 21% apresentaram risco para disfagia (EAT-10 ≥ 3 pontos ou queixa de dificuldades para deglutir e/ou mastigar) e tiveram a disfagia confirmada por meio da avaliação clínica da deglutição utilizando diferentes critérios, sendo um deles a FOIS < 7^(24)^. Não foram encontrados na literatura estudos que relacionaram os achados do EAT-10 e da FOIS em pacientes com diferentes tipos de neoplasias, como neste estudo.

O item do EAT-10 no qual os pacientes mais pontuaram algum grau de dificuldade foi o referente a fazer força para engolir remédios (Figura 1), com maior prevalência naqueles com risco para disfagia. Este achado corrobora um estudo que aplicou o EAT-10 com indivíduos não oncológicos e sem queixas de disfagia, em que 17% relataram algum grau de dificuldade para engolir remédios, sendo que dentre os participantes com risco para disfagia, 72% relataram esta dificuldade^(27)^. Outros estudos com pessoas hígidas também encontraram resultados semelhantes, com prevalência variando entre 10,4% e 32%^(28,29)^. Este dado é importante, pois indivíduos com dificuldades para engolir remédios podem descontinuar a medicação ou usar técnicas inapropriadas para manejar essas dificuldades, o que pode afetar a eficácia do medicamento^(28,30)^.

Entende-se que a presença de doenças orgânicas como acidente vascular encefálico, doenças neurodegenerativas e neoplasias, podem aumentar ainda mais a prevalência de disfagia entre os idosos^(30,31)^. Neste estudo, o risco para disfagia foi mais prevalente em pacientes idosos, corroborando com uma pesquisa com pacientes hospitalizados, que observou uma associação entre idade mais avançada e maior risco para disfagia (EAT-10), sendo que dos pacientes classificados com risco para disfagia, 61% tinham idade igual ou superior a 60 anos^(32)^. Em pacientes com câncer de laringe e hipofaringe observou-se que a disfagia era mais frequente em pacientes com idade igual ou superior a 65 anos^(33)^.

O presente estudo incluiu indivíduos com diferentes tipos de neoplasias, não somente tumores de cabeça e pescoço, além de ter boa representatividade em relação ao sexo, a idade e ao grupo etário (adultos e idosos). Como limitações do estudo, trata-se de uma amostra representativa de um perfil de pacientes de um local de referência no tratamento oncológico, sendo o seu tamanho estimado pelo número de leitos disponíveis. Além disso, optou-se por não realizar avaliação objetiva de deglutição, por conta das características da rotina do serviço. Espera-se que estudos futuros possam contemplar outras populações, maior amostragem e complementar as avaliações com exames objetivos de deglutição.

## CONCLUSÃO

O presente estudo demonstrou que quanto menor a pontuação no EAT-10, maior o nível da FOIS em pacientes oncológicos hospitalizados. Ou seja, quanto menor o risco para disfagia, menor a suscetibilidade ao uso de via alternativa de alimentação.

Esta correlação inversa entre o risco de disfagia e o nível de ingestão oral, sugere que quanto melhor a autopercepção do paciente em relação a sua deglutição, melhor a condição da alimentação via oral. Este achado, aponta para a relevância do uso de instrumentos de autoavaliação da deglutição na população com câncer em geral, visto que este tipo de instrumento de triagem promove a identificação rápida daqueles que necessitam de avaliação da deglutição especializada, propiciando diagnóstico e intervenção precoce.
